# Preoperative inflammatory markers as prognostic predictors after hepatocellular carcinoma resection: data from a western referral center

**DOI:** 10.1186/s12893-022-01779-6

**Published:** 2022-09-02

**Authors:** João Paulo Maciel Silva, Fabricio Ferreira Coelho, Alex Jones Flores Cassenote, Vagner Birk Jeismann, Gilton Marques Fonseca, Jaime Arthur Pirola Kruger, José Donizeti de Meira Júnior, Sérgio Carlos Nahas, Paulo Herman

**Affiliations:** 1 Instituto do Câncer do Estado de São Paulo (ICESP), Hospital das Clínicas (HCFMUSP), Faculdade de Medicina, Universidade de São Paulo, São Paulo, SP Brazil; 2 Instituto do Câncer do Estado de São Paulo (ICESP), Hospital das Clínicas (HCFMUSP), Faculdade de Medicina, Universidade de São Paulo, São Paulo, SP, BR Brazil; 3 Serviço de Cirurgia do Fígado, Divisão de Cirurgia do Aparelho Digestivo, Departamento de Gastroenterologia, Hospital das Clínicas (HCFMUSP), Faculdade de Medicina, Universidade de São Paulo, Av. Dr. Eneas de Carvalho Aguiar, 255, Cerqueira Cesar, São Paulo, SP 05403-000 Brazil

**Keywords:** Hepatectomy, Hepatocellular carcinoma, Inflammation, Prognosis, Survival analysis

## Abstract

**Background:**

Recent studies from eastern centers have demonstrate an association between inflammatory response and long-term outcomes after hepatocellular carcinoma (HCC) resection. However, the prognostic impact of inflammatory markers in western patients, with distinct tumor and epidemiologic features, is still unknown.

**Aim:**

To evaluate the prognostic impact of preoperative neutrophil-to-lymphocyte ratio (NLR), platelet-to-lymphocyte ratio (PLR), and monocyte-to-lymphocyte ratio (MLR), as well as their impact according to tumor size (< 5 cm, 5–10 cm, > 10 cm) in patients undergoing HCC resection with curative intent.

**Methods:**

Optimal cut-off values for NLR, PLR, and MLR were determined by plotting the receiver operator curves. Overall survival (OS) and disease-free survival (DFS) curves were calculated using the Kaplan–Meier method and compared using the log-rank test. The Cox method was used to identify independent predictors of OS and DFS.

**Results:**

In total, 161 consecutive adult patients were included. A high NLR (> 1.715) was associated with worse OS (P = 0.018). High NLR (> 2.475; P = 0.047) and PLR (> 100.25; P = 0.028) were predictors of short DFS. In HCC < 5 cm, MLR (> 1.715) was associated with worse OS (P = 0.047). In the multivariate analysis, high PLR was an independent predictor of worse DFS [hazard ratio (HR) 3.029; 95%CI 1.499–6.121; P = 0.002].

**Conclusion:**

Inflammatory markers are useful tools to predict long-term outcomes after liver resection in western patients, high NLR was able to stratify subgroups of patients with short OS and DFS, an increased PLR was an independent predictor of short DFS, while high MLR was associated with short OS in patients with early HCC.

**Supplementary Information:**

The online version contains supplementary material available at 10.1186/s12893-022-01779-6.

## Introduction

Hepatocellular carcinoma (HCC) is the third most frequent cause of cancer-associated mortality worldwide, with more than 900,000 deaths per year [1, 2]. Among the curative modalities, resection is one of the mainstays of HCC treatment; however, the recurrence rate remains high, reaching 50–80% in 5 years [3].

The main prognostic factors for patients with HCC who underwent resection are serum alpha-fetoprotein levels, the number of lesions, tumor size, and presence of vascular invasion and satellite nodules [4]. However, most of these factors can only be assessed after surgical specimen evaluation and cannot be used for preoperative patient selection. For this reason, the search for preoperative prognostic markers that may help understand the tumors’ biology is advisable.

Recent studies have shown an association between inflammatory response and long-term outcomes in several solid gastrointestinal tumors [5, 6]. However, the prognostic impact of inflammatory markers in patients who underwent surgical resection for HCC is still under debate.

The neutrophil-to-lymphocyte ratio (NLR) is the most studied preoperative biomarker for patients with HCC [7]. Moreover, recent studies have suggested that the NLR is also a prognostic factor in specific subgroups, such as patients with small tumors (< 5 cm) [8] or large HCCs (> 10 cm) [9]. However, other authors have failed to detect an association between NLR and HCC prognosis [10]. In recent years, a few eastern studies also suggested the impact of other inflammatory markers, such as the platelet-to-lymphocyte ratio (PLR) and monocyte-to-lymphocyte ratio (MLR), on long-term outcomes of HCC patients [11].

Despite promising outcomes, few studies conducted in western centers, where HCC presents distinct tumor and epidemiologic characteristics, have assessed the ability of NLR, PLR and MLR to predict long-term survival in patients with HCC undergoing liver resection [12]. Additionally, to our knowledge, no western studies have evaluated the impact of inflammatory markers on subsets of patients according to tumor size.

The primary endpoint of this study was to evaluate the prognostic impact of the NLR, PLR, and MLR on the long-term outcomes of patients who underwent curative hepatic resection for HCC. The secondary endpoint was to evaluate the prognostic impact of these markers on subgroups of patients according to tumor size: < 5 cm, 5–10 cm, and > 10 cm.

## Methods

This study was approved by the Institutional Ethics Committee of the Hospital das Clinicas, University of Sao Paulo School of Medicine (number: 3.004.022) and conducted according to the Standards for Reporting Studies of Diagnostic Accuracy (STARD) [13].

All methods were performed in accordance with the World Medical Association Declaration of Helsinki.

From a prospective database, consecutive adult patients with pathologically proven HCC who underwent liver resection with curative intent between January 2007 and December 2018 were evaluated. The inclusion criteria were as follows: patients older than 18 years, uni or oligonodular disease (up to three nodules), and absence of extrahepatic disease. Patients with chronic liver disease and compensated liver function were considered eligible as follows: Child–Pugh A (or B7 when minor peripheral resection was required), Model of End Stage Liver Disease (MELD) scores ≤ 10, and future liver remnant ≥ 40%. Portal hypertension was not an absolute contraindication for surgery, patients with small caliber esophageal varices and platelets > 100.000/mL were eligible when minor resection was required [14]. The exclusion criteria were presence of extrahepatic disease, R1/R2 resection, previous systemic or locoregional treatment addressed to HCC, presence of infection, and use of preoperative therapeutic antibiotics or corticosteroids.

All patients underwent clinical evaluation and laboratory tests for liver function. Preoperative workup included abdominal helicoidal computed tomography (CT) or magnetic resonance imaging (MRI), and thoracic CT. Preoperative diagnosis was based on image characteristics; biopsy was only indicated if diagnostic doubt persisted after radiologic evaluation. When CT or MRI showed signs of portal hypertension, upper digestive endoscopy was performed. Surgery was performed after a multidisciplinary meeting discussion.

The following preoperative characteristics were studied: age, sex, body mass index (BMI), preoperative laboratory tests, etiology of chronic liver disease, size and location of the lesions, presence of cirrhosis, and portal hypertension. Inflammatory markers were evaluated within 7 days of surgery. The NLR was calculated by dividing the absolute neutrophil count (number of neutrophils/mL) by the absolute lymphocyte count (number of lymphocytes/mL); the PLR was calculated by dividing the absolute platelet count (number of platelets/mL) by the absolute lymphocyte count (number of lymphocytes/mL); and the MLR was calculated by dividing the absolute monocyte count (number of monocytes/mL) by the absolute lymphocyte count (number of lymphocytes/mL).

For the intra- and postoperative periods, the following data were retrieved: blood transfusion requirement, length of stay in the intensive care unit (ICU), length of hospital stay, perioperative complications, overall survival (OS), and disease-free survival (DFS). The specimens obtained were assessed for the number of nodules, size of the larger nodule [in millimeters (mm)], degree of tumor differentiation (histological grade), presence of satellite lesions, and presence of vascular invasion.

Perioperative morbidity was defined as any event occurring during the first 90 postoperative days. OS was defined as the time interval between liver resection and the date of death or the most recent follow-up date if the patient was alive. DFS was defined as the time interval between liver resection and recurrence at any site (diagnosed on imaging or biopsy), the most recent follow-up date or death. Postoperative follow-up was performed using imaging and laboratory tests every 4 months for the first 2 years, and then annually.

### Statistical analysis

Continuous data were expressed as median and interquartile range [or 95% confidence interval (CI)] or mean ± standard deviation (SD). Categorical variables were expressed as percentages. Quantitative data were compared using the t-test or Mann–Whitney U-test, as appropriate. For categorical variables, Fisher’s exact test or the χ^2^ test was used. Statistical significance was set at 5%.

The optimal cut-off values for the NLR, PLR, and MLR were calculated using receiver operator curves (ROC) and Youden’s index. Thereafter, the patients were divided into two groups: below and above the calculated cut-offs. OS and DFS were estimated using the Kaplan–Meier method and compared using the log-rank test. Univariate and multivariate Cox proportional hazards models were used to identify predictors associated with OS and DFS. Variables with statistical significance (P < 0.05) on univariate analysis were included in the multivariate analysis.

## Results

### Baseline characteristics

During the study period, 207 patients with histologically confirmed diagnosis of HCC underwent liver resection; one patient (0.5%) was younger than 18 years, 12 (4.8%) patients underwent preoperative transarterial chemoembolization (TACE) or radiofrequency ablation, nine patients (4.3%) had a preoperative MELD > 10, 18 (8.7%) patients underwent R1 resections, and six (2.9%) presented signs of infection or the use of antibiotics immediately before the surgery. After applying the exclusion criteria, 161 patients were enrolled in the study. The baseline characteristics of the patients are summarized in Table 1. The main causes of chronic liver disease were hepatitis C (60%), hepatitis B (20%), nonalcoholic steatohepatitis (NASH, 11%), alcoholic liver disease (5%), and other etiologies (4%) and the median number of nodules was 1 ± 1.

**Table 1:** Baseline characteristics of the included patients (N = 161)

Age (years)	
Mean ± SD	62 ± 11
Median (min–max)	63 (18–86)
Sex (%)	
Male	108 (67.1%)
Female	53 (32.9%)
BMI (kg/m^2^)	
Mean ± SD	25.4 ± 4.5
Median (quartile 25–75)	24.9 (22.6–27.7)
Cirrhosis (%)	
Yes	135 (83.9%)
No	26 (16.1%)
Child–Pugh (%)^†^	
A5	111 (82.2%)
A6	17 (12.6%)
B7	7 (5.2%)
Preoperative MELD	
Mean ± SD	8 ± 3
Median (quartile 25–75)	8 (7–9)
Portal hypertension (%)	
Yes	43 (26.7%)
No	92 (73.3%)
Esophageal varices (%)	
Yes	22 (13.7%)
No	21 (86.3%)
Hemoglobin (g/dL)	
Mean ± SD	13.7 ± 3.8
Median (quartile 25–75)	13.7 (12.6–14.9)
Platelet count (/mm^3^)	
Mean ± SD	186,410 ± 97,208
Median (quartile 25–75)	170,000 (118,000–230,000)
Bilirubin (g/dL)	
Mean ± SD	0.72 ± 0.22
Median (quartile 25–75)	0.65 (0.47–0.89)
Aspartate aminotransferase (AST, U/L)	
Mean ± SD	62.0 ± 61.0
Median (quartile 25–75)	42.0 (28.0–68.0)
Alanine aminotransferase (ALT, U/L)	
Mean ± SD	54.7 ± 51.0
Median (quartile 25–75)	38.0 (25.0–69.0)
INR	
Mean ± SD	1.1 ± 0.1
Median (quartile 25–75)	1.1 (1.0–1.2)
Creatinine (mg/dL)	
Mean ± SD	1.0 ± 1.0
Median (quartile 25–75)	0.9 (0.7–1.1)
Alpha-fetoprotein (ng/mL)	
Mean ± SD	2483.1 ± 9906.5
Median (quartile 25–75)	19.0 (4.7–172.7)
Albumin (g/dL)	
Mean ± SD	4.0 ± 0.3
Median (quartile 25–75)	4.1 (3.7–4.5)
Neutrophil count (/mm^3^)	
Mean ± SD	3601 ± 3465
Median (quartile 25–75)	3300 (2300–4410)
Lymphocyte count (/mm^3^)	
Mean ± SD	1869 ± 773
Median (quartile 25–75)	1700 (1300–2300)
Monocyte count (/mm^3^)	
Mean ± SD	575 ± 308
Median (quartile 25–75)	510 (400–700)
NLR	
Mean ± SD	2.3 ± 2.2
Median (quartile 25–75)	1.9 (1.4–2.6)
PLR	
Mean ± SD	115.4 ± 89.4
Median (quartile 25–75)	96.2 (67.0–144.4)
MLR	
Mean ± SD	3.8 ± 2.0
Median (quartile 25–75)	3.5 (2.4–4.6)
Tumor size (mm)	
Mean ± SD	62.0 ± 50.7
Median (quartile 25–75)	42 (29.0–80.0)
Number of nodules	
Mean ± SD	1.23 ± 0.7
Median (quartile 25–75)	1.0 (1.0–1.0)
Tumor grade (%)	
Well differentiated	9 (5.6%)
Moderately differentiated	104 (64.6%)
Poor differentiated	28 (17.4%)
Unavailable	20 (12.4%)
Satellite nodules (%)	
Yes	40 (24.8%)
No	121 (75.2%)
Vascular invasion (%)^††^	
Yes	82 (50.9%)
No	75 (43.8%)
Unavailable	4 (2.5%)

*SD* standard deviation; *BMI* body mass index; *MELD* Model for End-Stage Liver Disease; *INR* international normalized ratio; *NLR* neutrophil-to-lymphocyte ratio; *PLR* platelet-to-lymphocyte ratio; *MLR* monocyte-to-lymphocyte ratio

^†^% of patients with cirrhosis

^††^Micro and macrovascular invasion histologically documented in the specimen

The median follow-up was 62 months. During the follow-up period 72 patients (44,7%) died and 75 recurred (47.2%). The median OS was 57 months (95%CI 35–78). The OS of the entire cohort was 65.2% at 3 years, 47.6% at 5 years and 28.4% at 10 years, while DFS was 61.1% at 3 years, 44.4% at 5 years and 20.1% at 10 years (Additional file 1: Fig. S1).

### Optimal cut-offs for NLR, PLR and MLR

The cut-off values of the inflammatory markers were determined by plotting the ROC curves for mortality and recurrence after resection. The best cut-offs calculated using the Youden index are listed in Table 2.

**Table 2:** Diagnostic accuracy of the calculated cut-offs for mortality and recurrence

	Cut-off	Sensibility	Specificity	1—Specificity	LR+	LR−
Mortality						
NLR	> 1.715	0.639	0.483	0.517	1.236	0.747
PLR	> 115.050	0.375	0.697	0.303	1.236	0.897
MLR	> 1.750	0.917	0.112	0.888	1.033	0.742
Recurrence						
NLR	> 2.475	0.307	0.732	0.268	1.146	0.947
PLR	> 100.250	0.520	0.620	0.380	1.367	0.775
MLR	> 2.680	0.747	0.310	0.690	1.082	0.818

The NLR, PLR, and MLR areas under the curve (AUC) for mortality were 0.541 (95%CI 0.451–0.631), 0.479 (95%CI 0.388–0.571), and 0.454 (95%CI 0.365–0.543), respectively. Regarding recurrence, the calculated AUC were 0.479 (95%CI 0.385–0.573), 0.519 (95%CI 0.424–0.614), and 0.469 (95%CI 0.372–0.565), respectively

*NLR* neutrophil-to-lymphocyte ratio; *PLR* platelet-to-lymphocyte ratio; MLR monocyte-to-lymphocyte ratio; *LR+* positive likelihood ratio; *LR−* negative likelihood ratio

### Prognostic value of inflammatory markers for OS and DFS

**Fig. 1:**
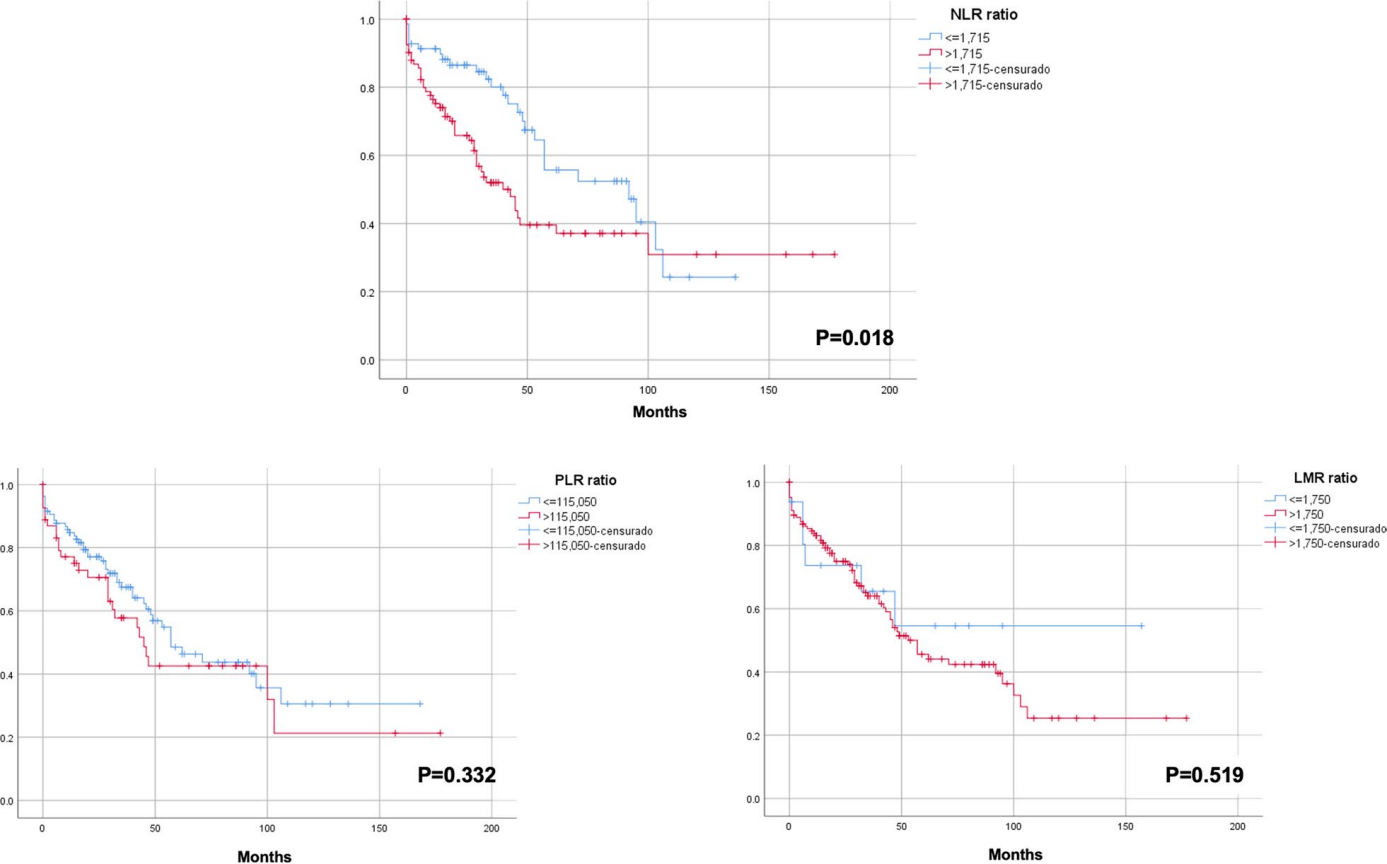
Overall survival of hepatocellular carcinoma patients with low (blue) and high (red) neutrophil-to-lymphocyte ratio (NLR), platelet-to-lymphocyte ratio (PLR), and monocyte-to-lymphocyte ratio (MLR)

A high NLR (> 1.715) was associated with short OS in patients who underwent HCC resection. The median OS in the subgroup of patients with high and low NLR were 40 months (95%CI 25–54) and 92 months (95%CI 49–120), respectively. The 5-year OS was 56% in the low NLR group and 40% in the high NLR group (P = 0.018, Fig. 1).

Clinicopathological characteristics of patients with low (≤ 1.715) and high NLR (> 1.715) are summarized in Additional file 1: Table S1. Patients with high NLR had lower serum albumin levels [4.1 g/dL (3.7–4.5) vs. 4.3 g/dL (4.1–4.6); P = 0.028] and larger tumors [77 mm (35–100) vs. 39 mm (21–45); P < 0.001] and were associated with higher values of PLR [134 (91.2–160) vs. 72.4 (53.7–93.2); P < 0.001] and MLR [4.4 (3.4–5.5) vs. 3.1 (2–3.8); P < 0.001].

**Fig. 2:**
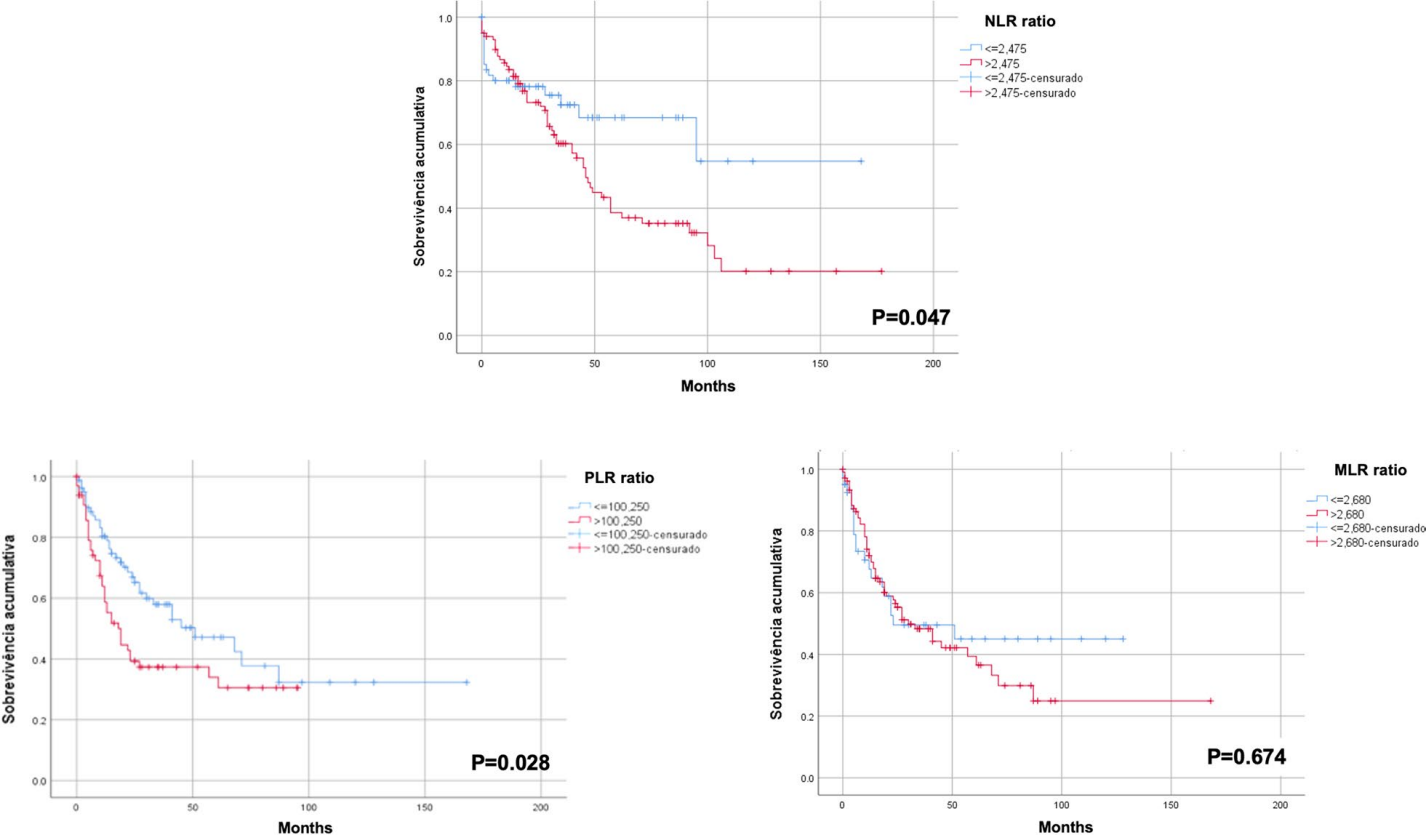
Disease-free survival of hepatocellular carcinoma patients with low (blue) and high (red) neutrophil-to-lymphocyte ratio (NLR), platelet-to-lymphocyte ratio (PLR), and monocyte-to-lymphocyte ratio (MLR)

High NLR (> 2.475) and PLR (> 100.25) were associated with short DFS in HCC patients treated with hepatectomy (Fig. 2).

Patients with high NLR (> 2.475) presented higher total bilirubin levels [0.7 g/dL (0.5–0.9) vs. 0.6 g/dL (0.5–0.7); P = 0.020] and larger tumors [67 mm (40–100) vs. 40 mm (25–65); P = 0.003] when compared to patients with low NLR. There was also an association with high PLR [147.2 (104.5–176) vs. 82.3 (60–108); P < 0.001] and high MLR [3.8 (3–5.2) vs. 2.1 (1.6–3.3); P < 0.001] (Additional file 1: Table S2).

Patients with high PLR (> 100.25) presented higher serum levels of total bilirubin [0.7 g/dL (0.5–0.9) vs. 0.6 g/dL (0.4–0.8); P = 0.004], larger tumors [75 mm (40–125) vs. 34 mm (22–45); P < 0.001], and a higher frequency of vascular invasion (62.1% vs. 42%; P = 0.020). Additionally, an association with higher values of NLR [2.5 (1.9–3.5) vs. 2.5 (1.9–3.6); P < 0.001] and MLR [4 (3–5.2) vs. 3.1 (1.8–3.8); P < 0.001] were observed (Additional file 1: Table S3).

### Risk factors for OS and DFS after hepatectomy

All clinicopathological and surgical characteristics were included in the univariate analysis. Variables associated with OS and DFS after HCC resection on univariate and multivariate analysis are shown in Table 3.

**Table 3:** Univariate and multivariate analyses of prognostic factors associated with overall and disease-free survival

Overall survival			Disease-free survival		
Variable	P	HR IC95%	Variable	P	HR IC95%
Univariate analysis					
Hepatitis C	0.016	1.98 (1.13–3.40)	Satellites nodules	0.023	1.77 (1.08–2.92)
Portal hypertension	0.005	2.16 (1.25–3.74)	Vascular invasion	0.005	1.97 (1.22–3.19)
Esophageal varices	0.047	1.90 (1.10–3.60)	Age > 50 years	0.050	0.54 (0.95–1.00)
Transfusion	0.002	2.38 (1.38–4.10)	Bilirubin > 1.2 mg/dL	0.034	2.41 (1.15–5.07)
Perioperative complications	0.006	2.00 (1.21–3.34)	AST > 50 U/dL	0.020	1.63 (1.10–2.59)
Vascular invasion	0.007	2.02 (1.20–3.40)	Alpha-fetoprotein > 20 ng/mL	< 0.001	3.64 (2.23–5.91)
Bilirubin > 1.2 mg/dL	0.048	1.90 (1.05–3.60)	NLR > 2.475	0.047	1.28 (1.01–1.96)
AST > 50 U/dL	0.021	1.85 (1.09–3.14)	PLR > 100.25	0.028	1.60 (1.02–2.52)
ICU stay > 3 days	< 0.001	3.06 (1.80–5.23)			
Alpha-fetoprotein > 20 ng/mL	< 0.001	3.42 (1.96–5.91)			
NLR > 1.715	0.018	1.61 (1.01–2.67)			
Multivariate analysis					
Portal hypertension	< 0.001	7.04 (2.40-20.66)	Vascular invasion	0.022	2.36 (1.13–4.93)
Vascular invasion			AST > 50 ng/mL	0.001	3.32 (1.60–6.91)
AST > 50 U/dL	0.032	3.06 (1.10–8.47)	PLR > 100.25	0.002	3.03 (1.50–6.12)
ICU stay > 3 days	0.003	5.04 (1.75–14.49)			

Multivariate analysis showed that the presence of portal hypertension, preoperative aspartate aminotransferase, and ICU stay > 3 days were independent predictors of short OS. Regarding DFS, AST level > 50 U/dL presence of vascular invasion and high PLR were predictors of a high recurrence rate

*AST* aspartate aminotransferase; *ICU* intensive care unit; *NLR* neutrophil-to-lymphocyte ratio; *PLR* platelet-to-lymphocyte ratio

### Subgroup analysis

Survival analysis was also performed in patients with HCC according to tumor size: < 5 cm (group 1, N = 98), 5–10 cm (group 2, N = 35), and > 10 cm (group 3, N = 28).

A high MLR (> 1.750) was associated with short OS in group 1 (P = 0.047) (Additional file 1: Fig. S2). None of the inflammatory markers were associated with DFS in this subset of patients (Additional file 1: Fig. S3). In groups 2 and 3, the NLR, PLR, and MLR were not associated with OS or DFS (Additional file 1: Figs. S4–S7).

## Discussion

Systemic inflammatory status has impact on carcinogenesis [15]. Recent studies have shown that the molecular environment created by humoral response favors conjunctive matrix degradation, neoangiogenesis, and activation of cell profiles favoring tissue invasion and metastatic dissemination. Therefore, an increase in humoral inflammatory response can lead to worse oncological outcomes [15]. Conversely, lymphocytic cellular response (mediated by T lymphocytes CD4+, CD8+, and NK cells) inhibits carcinogenesis, leading to better oncological prognosis [16]. Recent studies have also shown the interaction between platelets and tumoral microenvironment [17]. The main platelet-associated mechanisms are based on signaling pathways that orchestrate tumor growth, activation of angiogenesis, and metastatic dissemination [18].

The prognostic impact of systemic inflammatory response has been studied in several gastrointestinal tumors, such as pancreatic, colorectal, and gastric cancers [19, 20]. The main advantages of inflammatory markers include calculation using routine laboratory tests, low cost, and access to results before therapeutic intervention [21].

The NLR is the most studied inflammatory index. A large metanalysis, comprising more than 40,000 patients showed an association of high NLR with lower survival rate in patients with several solid tumors [22].

However, the prognostic impact of inflammatory markers in patients with HCC who undergo resection remains controversial. Most studies that assessed these prognostic markers came from eastern centers, where HCC presents distinct clinical and epidemiological features [23]. The present study is one of the first from a western center to evaluate the association between the main inflammatory markers (NLR, PLR, and MLR) and long-term outcomes after liver resection for HCC. In our study, the mean age was 62 ± 11 years, similar to those in other western centers but higher than those in eastern centers (52 ± 9 years) [24]. Regarding chronic liver disease etiology, hepatitis C (60%) was the most frequent, followed by hepatitis B (20%) and NASH (11%). In contrast, in eastern centers, the prevalence of hepatitis B infection is higher than 50% [25]. Our data showed that 84% of patients had chronic liver disease and 94.8% were classified as Child–Pugh A. Beard et al. [26] compared surgical outcomes after HCC resection in cirrhotic and non-cirrhotic North American patients and found a cirrhosis prevalence of 73%. In the eastern centers, the prevalence of cirrhosis/chronic liver disease is lower than 54% [8].

The preoperative NLR is the most studied biomarker in patients with HCC. Although several studies have suggested that high NLR may correlate with a poor prognosis [7, 8], others failed to detect this association [10]. Furthermore, it is important to point out the wide heterogeneity regarding the cut-off values across the studies. Wang et al. [23] in a recent meta-analysis, included 17 studies (13 for OS and 7 for DFS) finding cut-off values for NLR ranging from 1.51 to 5.0. Moreover, most of the studies are from eastern centers, and use the same cut-off for OS and DFS [23, 27].

The present study showed that NLR > 1.715 and > 2.745 were associated with short OS and DFS in univariate analysis, respectively. A recent meta-analysis conducted by Xingshun et al. [28] including 20,475 patients with HCC (90 studies) who underwent different treatments (liver transplant, liver resection, ablation, and sorafenib) found that low baseline NLR was significantly associated with better OS (HR 1.80, 95%CI 1.59–2.04, P < 0.00001) and DFS (HR 2.23, 95% CI 1.80–2.76, P < 0.00001). In the subgroup of patients who underwent liver resection (12 studies, 3097 patients) low baseline NLR was also associated with better OS (HR 1.95, 95%CI 1.61–2.37, P < 0.00001) and DFS (HR 1.87, 95%CI 1.47–2.37, P < 0.00001).

However, in the multivariate analysis, the NLR was not an independent factor associated with OS or DFS in our study, which was also observed in other studies, especially from western centers. Sullivan et al. [10] evaluating patients with HCC found that the NLR was not a predictor for OS after surgical or locoregional treatment (HR 1.09; 95%CI 0.95–1.24; P = 0.23). Another study from the United Kingdom showed that the NLR was a predictor of DFS (HR 4.67; 95%CI 1.88–11.64; P = 0.001) but not a predictor of OS in cirrhotic patients undergoing HCC resection. Interestingly, no relationship was found between NLR and prognosis in non-cirrhotic patients [29]. Thus, the presence of cirrhosis may impact the predictive value of NLR, justifying the heterogeneous results between the available studies.

Few studies have addressed the prognostic impact of other inflammatory markers in HCC patients [30]. In our study, we observed that high PLR (> 100.25) was an independent factor of shorter DFS, which is consistent with recent studies [31]. Kaida et al. [32] evaluated patients with early-stage HCC who underwent resection and compared five inflammatory marker scores, showing that preoperative PLR was an independent predictor of recurrence. Similarly, Qing et al. [31] showed that increased preoperative platelet levels were associated with a higher recurrence rate following HCC resection. To date, few studies have evaluated the prognostic impact of MLR in HCC patients [33].

Recent studies have suggested that the inflammatory markers are also prognostic factors in specific subgroups, such as patients with small tumors (< 5 cm) [8] or large HCCs (> 10 cm) [9]. Historically, size is a main prognostic factor for HCC patients. Well-established staging systems such as TNM, Milan criteria and Barcelona Clinic Liver Cancer (BCLC) included tumor size in therapeutic algorithm and prognostic stratification. In fact, 5 cm is a landmark in TNM staging (T2 vs. T3), Milan criteria and BLCL (early HCC). Additionally, some authors showed worse prognosis in patients with HCC > 10 cm (called large or huge HCCs). Based on these data, we stratified our patients according to tumor size (< 5 cm, 5–10 cm, and > 10 cm). An interesting finding of our study was the association of low MLR with better OS in patients with early-stage HCC (< 5 cm). This finding can be justified by the fact that activation of monocytes and macrophages usually occurs at earlier stages of tumor growth. Otherwise, in patients with larger lesions, other cells such as neutrophils and platelets play a predominant role in local invasion and metastatic dissemination [34].

Another independent factor associated with short OS in the present study was the presence of portal hypertension, which is in accordance with other studies [35]. In a meta-analysis comprising 2285 patients with HCC who underwent resection, the group of patients with portal hypertension presented short OS than the group without portal hypertension (HR 1.48; 95%CI 1.11–1.98; P = 0.007) [36]. An AST level > 50 U/dL was an independent factor related to both OS and DFS. The exact mechanism underlying this finding is poorly understood; however, it might be explained by the fact that AST is exclusively present in hepatocytes and released into the circulation during liver inflammatory insults. Additionally, the reduced clearance in progressive chronic hepatic disease can lead to an increase in AST levels [37]. In our study, microvascular invasion was also an independent prognostic factor for recurrence. In fact, vascular invasion is frequently associated with higher recurrence rates due to aggressive biological behavior, represented by a greater volume of micrometastatic disease and a higher frequency of mural invasion [38].

Based on our findings, all the studied inflammatory markers are useful tools to predict long-term outcomes after liver resection in western patients. High NLR was able to stratify subgroups of patients with short OS and DFS, and increased PLR was a marker of short DFS, while high MLR was associated with short OS in patients with early HCC. In fact, these markers were able to identify subgroups of patients with poor clinical features, such as higher bilirubin levels, larger tumors, and a higher frequency of vascular invasion. Therefore, inflammatory indexes are promising tools for preoperative selection of patients who require strict postoperative follow-up or even potential candidates for new adjuvant strategy protocols.

However, our findings should be viewed with caution due to some limitations. The first was the retrospective nature of this study, which increases the risk of selection, confusion, and measurement biases. Another limitation was the small number of patients enrolled, which may impair statistical power, especially in the subgroup analysis. Thus, the insights provided herein should be confirmed by larger prospective studies.

In conclusion, our study suggested that a high preoperative NLR is associated with short OS and DFS, whereas a high PLR is an independent factor associated with short DFS. In the subset of patients with HCC < 5 cm, a high MLR is associated with short OS.

## Supplementary Information

**Additional file 1: Figure S1.** Overall and disease-free survival of patients with hepatocellular carcinoma included in the study (N=161). **Figure S2.** Overall survival of patients with hepatocellular carcinoma < 5 cm (Group 1) with low (blue) and high (red) neutrophil-to-lymphocyte ratio (NLR), platelet-to-lymphocyte ratio (PLR), and monocyte-to-lymphocyte ratio (MLR). **Figure S3.** Disease-free survival of patients with hepatocellular carcinoma < 5 cm (Group 1) with low (blue) and high (red) neutrophil-to-lymphocyte ratio (NLR), platelet-to-lymphocyte ratio (PLR), and monocyte-to-lymphocyte ratio (MLR). **Figure S4.** Overall survival of patients with hepatocellular carcinoma between 5 and 10 cm (Group 2) with low (blue) and high (red) neutrophil-to-lymphocyte ratio (NLR), platelet-to-lymphocyte ratio (PLR), and monocyte-to-lymphocyte ratio (MLR). **Figure S5.** Disease-free survival of patients with hepatocellular carcinoma between 5 and 10 cm (Group 2) with low (blue) and high (red) neutrophil-to-lymphocyte ratio (NLR), platelet-to-lymphocyte ratio (PLR), and monocyte-to-lymphocyte ratio (MLR). **Figure S6.** Overall survival of patients with hepatocellular carcinoma > 10 cm (Group 3) with low (blue) and high (red) neutrophil-to-lymphocyte ratio (NLR), platelet-to-lymphocyte ratio (PLR), and monocyte-to-lymphocyte ratio (MLR). **Figure S7.** Disease-free survival of patients with hepatocellular carcinoma > 10 cm (Group 3) with low (blue) and high (red) neutrophil-to-lymphocyte ratio (NLR), platelet-to-lymphocyte ratio (PLR), and monocyte-to-lymphocyte ratio (MLR). **Table S1.** Baseline characteristics of patients with low (≤ 1.715) and high (> 1.715) neutrophil-to-lymphocyte ratio (NLR). **Table S2.** Baseline characteristics of patients with low (≤ 2.475) and high (> 2.475) neutrophil-to-lymphocyte ratio (NLR). **Table S3.** Baseline characteristics of patients with low (≤ 100.25) and high (> 100.25) platelet-to-lymphocyte ratio (PLR)

## Data Availability

The data that support the findings of this study are available from the corresponding author (J.P.M.S), upon reasonable request.

## Abbreviations

HCC: Hepatocellular carcinoma
NK: Natural killer cells
NLR: Neutrophil-to-lymphocyte ratio
PLR: Platelet-to-lymphocyte ratio
MLR: Monocyte-to-lymphocyte ratio
STARD: Standards for Reporting Studies of Diagnostic Accuracy
MELD: Model for End-Stage Liver Disease
CT: Computed tomography scan
MRI: Magnetic resonance imaging
BMI: Body mass index
ICU: Intensive care unit
OS: Overall survival
DFS: Disease-free survival
ROC: Receiver operator curves
TACE: Transarterial chemoembolization
NASH: Non-alcoholic steatohepatitis
SD: Standard deviation
AST: Aspartate aminotransferase
ALT: Alanine aminotransferase
INR: Interna tional normalized ratio
LR+: Positive likelihood ratio
LR−: Negative likelihood ratio
AUC: Area under the curve
