# Supplementary material for: Preoperative inflammatory markers as prognostic predictors after hepatocellular carcinoma resection: data from a western referral center
Source: BMC Surg. 2022 Sep 2;22:329. doi: 10.1186/s12893-022-01779-6 (PMC9440527; doi:10.1186/s12893-022-01779-6)
Supplement: Supplementary file 1 — Additional file 1: Figure S1. Overall and disease-free survival of patients with hepatocellular carcinoma included in the study (N=161). Figure S2. Overall survival of patients with hepatocellular carcinoma < 5 cm (Group 1) with low (blue) and high (red) neutrophil-to-lymphocyte ratio (NLR), platelet-to-lymphocyte ratio (PLR), and monocyte-to-lymphocyte ratio (MLR). Figure S3. Disease-free survival of patients with hepatocellular carcinoma < 5 cm (Group 1) with low (blue) and high (red) neutrophil-to-lymphocyte ratio (NLR), platelet-to-lymphocyte ratio (PLR), and monocyte-to-lymphocyte ratio (MLR). Figure S4. Overall survival of patients with hepatocellular carcinoma between 5 and 10 cm (Group 2) with low (blue) and high (red) neutrophil-to-lymphocyte ratio (NLR), platelet-to-lymphocyte ratio (PLR), and monocyte-to-lymphocyte ratio (MLR). Figure S5. Disease-free survival of patients with hepatocellular carcinoma between 5 and 10 cm (Group 2) with low (blue) and high (red) neutrophil-to-lymphocyte ratio (NLR), platelet-to-lymphocyte ratio (PLR), and monocyte-to-lymphocyte ratio (MLR). Figure S6. Overall survival of patients with hepatocellular carcinoma > 10 cm (Group 3) with low (blue) and high (red) neutrophil-to-lymphocyte ratio (NLR), platelet-to-lymphocyte ratio (PLR), and monocyte-to-lymphocyte ratio (MLR). Figure S7. Disease-free survival of patients with hepatocellular carcinoma > 10 cm (Group 3) with low (blue) and high (red) neutrophil-to-lymphocyte ratio (NLR), platelet-to-lymphocyte ratio (PLR), and monocyte-to-lymphocyte ratio (MLR). Table S1. Baseline characteristics of patients with low (≤ 1.715) and high (> 1.715) neutrophil-to-lymphocyte ratio (NLR). Table S2. Baseline characteristics of patients with low (≤ 2.475) and high (> 2.475) neutrophil-to-lymphocyte ratio (NLR). Table S3. Baseline characteristics of patients with low (≤ 100.25) and high (> 100.25) platelet-to-lymphocyte ratio (PLR) [file 12893_2022_1779_MOESM1_ESM.docx]

**Additional material**

**
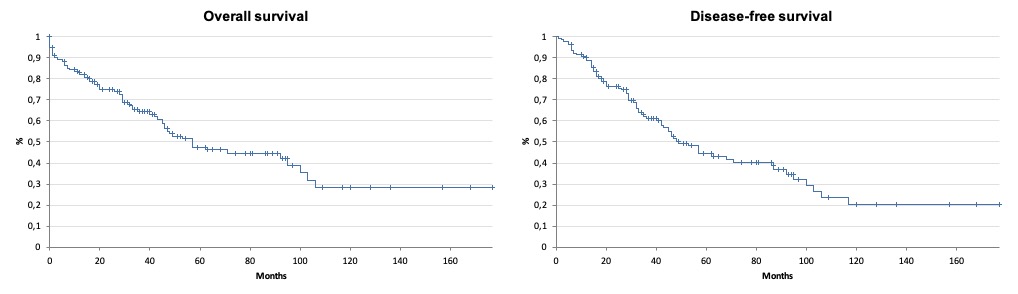
**

**Figure S1.** Overall and disease-free survival of patients with hepatocellular carcinoma included in the study (N=161)

**Table S1.** Baseline characteristics of patients with low (≤ 1.715) and high (> 1.715) neutrophil-to-lymphocyte ratio (NLR)

|  | NLR | |  |
| --- | --- | --- | --- |
|  | **≤ 1.715** | **> 1.715** | **P** |
| Age (years)  mean ± SD  median (min-max) | 61 ± 9  63 (18-79) | 63 ± 12  64 (20-86) | 0.209 |
| Gender (%)  male  female | 39 (60.9%)  25 (39.1%) | 69 (71.1%)  28 (28.9%) | 0.230 |
| Cirrhosis (%)  yes  no | 5 (7.8%)  59 (92.2%) | 15 (15.4%)  82 (50.6%) | 0.122 |
| Portal hypertension (%)  yes  no | 34 (53.1%)  30 (46.9%) | 49 (50.5%)  48 (49.5%) | 0.748 |
| Esophageal varices (%)  yes  no | 9 (14.1%)  55 (85.9%) | 13 (13.4%)  84 (86.6%) | 1 |
| Child-Pugh (%)^†^  A5  A6  B7 | 49 (74.6%)  14 (21.8%)  1 (1.6%) | 62 (57.3%)  30 (30.9%)  5 (5.2%) | 0.117 |
| MELD  mean ± SD  median (quartile 25-75) | 8 ± 2  8 (7-9) | 9 ± 3  9 (7-10) | 0.159 |
| Bilirubin (g/dL)  mean ± SD  median (quartile 25-75) | 0.7 ± 0.1  0.6 (0.5-0.9) | 0.7 ± 0.3  0.6 (0.5-0.9) | 0.862 |
| Aspartate aminotransferase (AST. U/L)  mean ± SD  median (quartile 25-75) | 60 ± 46  47 (29-72) | 62 ± 32  42 (27-64) | 0.671 |
| Alanine aminotransferase (ALT. U/L)  mean ± SD  median (quartile 25-75) | 56 ± 50  41 (30-69) | 51 ± 51  35 (21-59) | 0.068 |
| Alpha-fetoprotein (ng/mL)  mean ± SD  median (quartile 25-75) | 382 ± 897.5  21.6 (5.1-227.7) | 3975.6 ± 15975  22 (4.9-151) | 0.784 |
| Albumin (g/dL)  mean ± SD  median (quartile 25-75) | 4.2 ± 0.3  4.3 (4.1-4.6) | 4.0 ± 0.3  4.1 (3.7-4.5) | **0.028** |
| PLR  mean ± SD  median (quartile 25-75) | 89.9 ± 103  72.4 (53.7-93.2) | 134 ± 73.1  111.4 (91.2-160) | **<0.001** |
| MLR  mean ± SD  median (quartile 25-75) | 3.2 ± 3.9  3.1 (2-3.8) | 4.6 ± 3.3  4.4 (3.4-5.5) | **<0.001** |
| Tumor size (mm)  mean ± SD  median (quartile 25-75) | 39 ± 26  35 (21-45) | 77 ± 57  58 (35-100) | **<0.001** |
| Vascular invasion  yes  no | 32 (50%)  32 (50%) | 43 (44.3%)  54 (55.6%) | 0.521 |

SD: standard deviation; MELD: Model for End-Stage Liver Disease; PLR: platelet-to-lymphocyte ratio; MLR: monocyte-to-lymphocyte ratio

^†^ % of patients with cirrhosis

**Table S2.** Baseline characteristics of patients with low (≤ 2.475) and high (> 2.475) neutrophil-to-lymphocyte ratio (NLR)

|  | NLR | |  |
| --- | --- | --- | --- |
|  | **≤ 2.475** | **> 2.475** | **P** |
| Age (years)  mean ± SD  median (min-max) | 62 ± 11  64 (18-86) | 63 ± 10  61 (19-79) | 0.773 |
| Gender (%)  male  female | 69 (63.3%)  40 (36.7%) | 33 (78.6%)  9 (21.4%) | 0.083 |
| Cirrhosis (%)  yes  no | 91 (83.5%)  18 (16.5%) | 36 (85.7%)  6 (14.3%) | 0.122 |
| Portal hypertension (%)  yes  no | 28 (25.7%)  57 (74.3%) | 11 (30.6%)  25 (69.4%) | 0.835 |
| Esophageal varices (%)  yes  no | 15 (13.8%)  94 (86.2%) | 7 (16.7%)  35 (83.3%) | 0.617 |
| Child-Pugh (%)^†^  A5  A6  B7 | 76 (84.6%)  11 (12.1%)  3 (3.3%) | 30 (88.3%)  4 (11.1%)  2 (5.6%) | 0.366 |
| MELD  mean ± SD  median (quartile 25-75) | 8 ± 2  8 (7-9) | 9 ± 3  8 (7-10) | 0.388 |
| Bilirubin (g/dL)  mean ± SD  median (quartile 25-75) | 0.6 ± 0.3  0.6 (0.5-0.7) | 0.8 ± 0.4  0.7 (0.5-0.9) | **0.020** |
| Aspartate aminotransferase (AST. U/L)  mean ± SD  median (quartile 25-75) | 59 ± 59  44 (29-66) | 65 ± 65  40 (24-74) | 0.470 |
| Alanine aminotransferase (ALT. U/L)  mean ± SD  median (quartile 25-75) | 56 ± 53  41 (27-64) | 53 ± 47  38 (20-80) | 0.491 |
| Alpha-fetoprotein (ng/mL)  mean ± SD  median (quartile 25-75) | 2051.4 ± 9625.9  16.2 (4.6-141.4) | 2455 ± 7045.6  35.4 (4.7-424.3) | 0.338 |
| Albumin (g/dL)  mean ± SD  median (quartile 25-75) | 4.1 ± 0.6  4.1 (3.8-4.5) | 4.0 ± 0.6  4.0 (3.6-4.5) | 0.577 |
| PLR  mean ± SD  median (quartile 25-75) | 98 ± 87.5  82.3 (60-108) | 157 ± 81.9  147.2 (104.5-176) | **<0.001** |
| MLR  mean ± SD  median (quartile 25-75) | 2.5 ± 1.1  2.1 (1.6-3.3) | 4.4 ± 2.1  3.8 (3-5.2) | **<0.001** |
| Tumor size (mm)  mean ± SD  median (quartile 25-75) | 54 ± 44  40 (25-65) | 80 ± 58  67 (40-100) | **0.003** |
| Vascular invasion  yes  no | 51 (48.6%)  54 (51.4%) | 24 (57.1%)  18 (42.9%) | 0.367 |

SD: standard deviation; MELD: Model of End Stage Liver Disease; PLR: platelet-to-lymphocyte ratio; MLR: monocyte-to-lymphocyte ratio

^†^ % of patients with cirrhosis

**Table S3.** Baseline characteristics of patients with low (≤ 100.25) and high (> 100.25) platelet-to-lymphocyte ratio (PLR)

|  | PLR | |  |
| --- | --- | --- | --- |
|  | **≤ 100.25** | **> 100.25** | **P** |
| Age (years)  mean ± SD  median (min-max) | 63 ± 9  64 (18-81) | 60 ± 13  60 (19-86) | 0.112 |
| Gender (%)  male  female | 53 (63.1%)  31 (36.9%) | 49 (73.1%)  18 (26.9%) | 0.223 |
| Cirrhosis (%)  yes  no | 75 (83.3%)  9 (10.7%) | 52 (74.6%)  15 (22.4%) | 0.072 |
| Portal hypertension (%)  yes  no | 25 (29.8%)  59 (70.2%) | 14 (20.9%)  53 (79.1%) | 0.431 |
| Esophageal varices (%)  yes  no | 14 (16.7%)  70 (83.3%) | 8 (11.9%)  59 (88.1%) | 0.490 |
| Child-Pugh (%)^†^  A5  A6  B7 | 63 (84%)  10 (13.3%)  2 (2.7%) | 46 (88,5%)  3 (5.8%)  3 (5.8%) | 0.351 |
| MELD  mean ± SD  median (quartile 25-75) | 8 ± 2  8 (7-9) | 9 ± 3  8 (7-9) | 0.881 |
| Bilirubin (g/dL)  mean ± SD  median (quartile 25-75) | 0.7 ± 0.4  0.6 (0.4-0.8) | 0.8 ± 0.4  0.7 (0.5-0.9) | **0.004** |
| Aspartate aminotransferase (AST, U/L)  mean ± SD  median (quartile 25-75) | 63 ± 66  44 (29-68) | 59 ± 53  40 (26-66) | 0.495 |
| Alanine aminotransferase (ALT. U/L)  mean ± SD  median (quartile 25-75) | 57 ± 46  46 (28-67) | 53 ± 58  34 (21-69) | 0.093 |
| Alpha-fetoprotein (ng/mL)  mean ± SD  median (quartile 25-75) | 489 ± 1782.7  18.8 (5.8-174.4) | 4278.8 ± 13149.5  19 (2.6-161) | 0.390 |
| Albumin (g/dL)  mean ± SD  median (quartile 25-75) | 4.1 ± 0.6  4.1 (3.8-4.5) | 4.1 ± 0.5  4.1 (3.7-4.5) | 0.889 |
| NLR  mean ± SD  median (quartile 25-75) | 1.6 ± 0.7  1.5 (1.1-2.0) | 3.2 ± 3.1  2.5 (1.9-3.6) | **<0.001** |
| MLR  mean ± SD  median (quartile 25-75) | 3.4 ± 2.4  3.1 (1.8-3.8) | 4.2 ± 1.6  4 (3-5.2) | **<0.001** |
| Tumor size (mm)  mean ± SD  median (quartile 25-75) | 41 ± 28  34 (22-45) | 86 ± 59  75 (40-125) | **<0.001** |
| Vascular invasion  yes  no | 34 (42%)  47 (58%) | 41 (62.1%)  25 (37.9%) | **0.020** |

SD: standard deviation; MELD: Model for End-Stage Liver Disease; NLR: neutrophil-to-lymphocyte ratio; MLR: monocyte-to-lymphocyte ratio

^†^ % of patients with cirrhosis


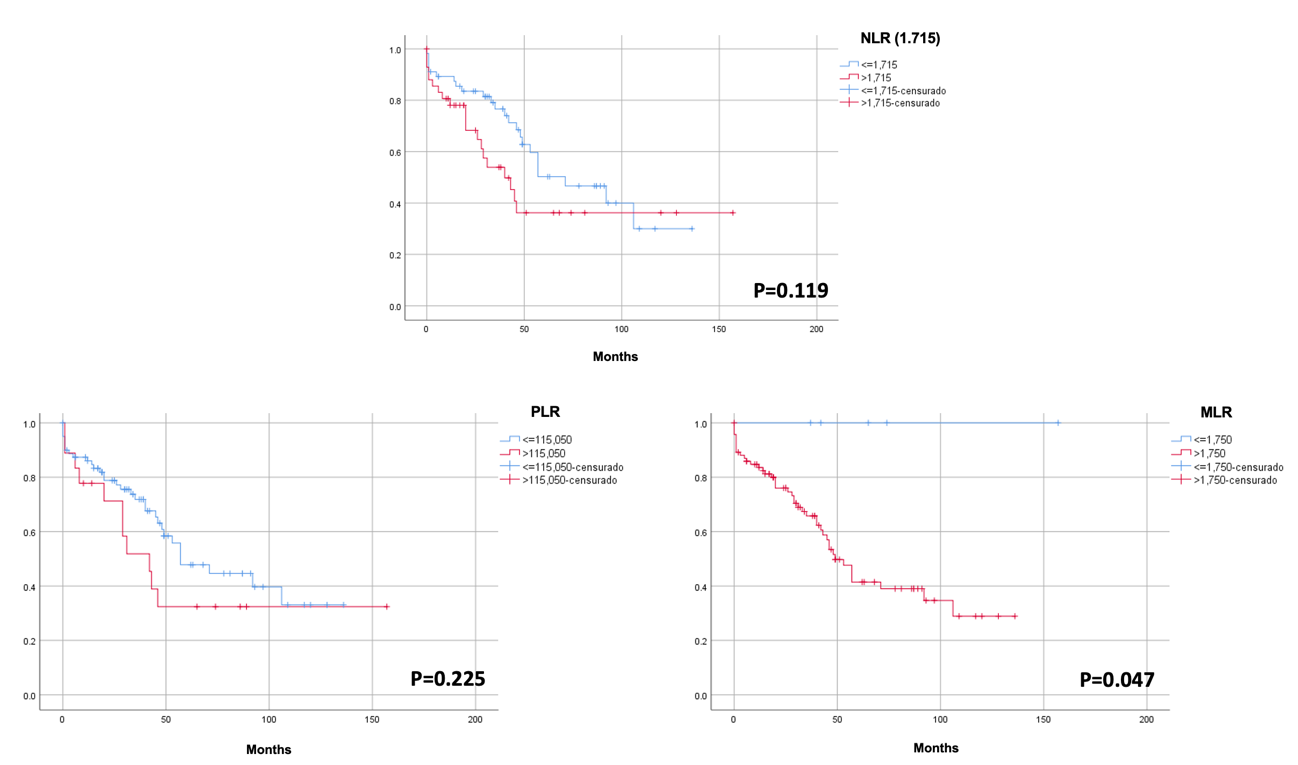


**Figure S2.** Overall survival of patients with hepatocellular carcinoma < 5 cm (Group 1) with low (blue) and high (red) neutrophil-to-lymphocyte ratio (NLR), platelet-to-lymphocyte ratio (PLR), and monocyte-to-lymphocyte ratio (MLR)


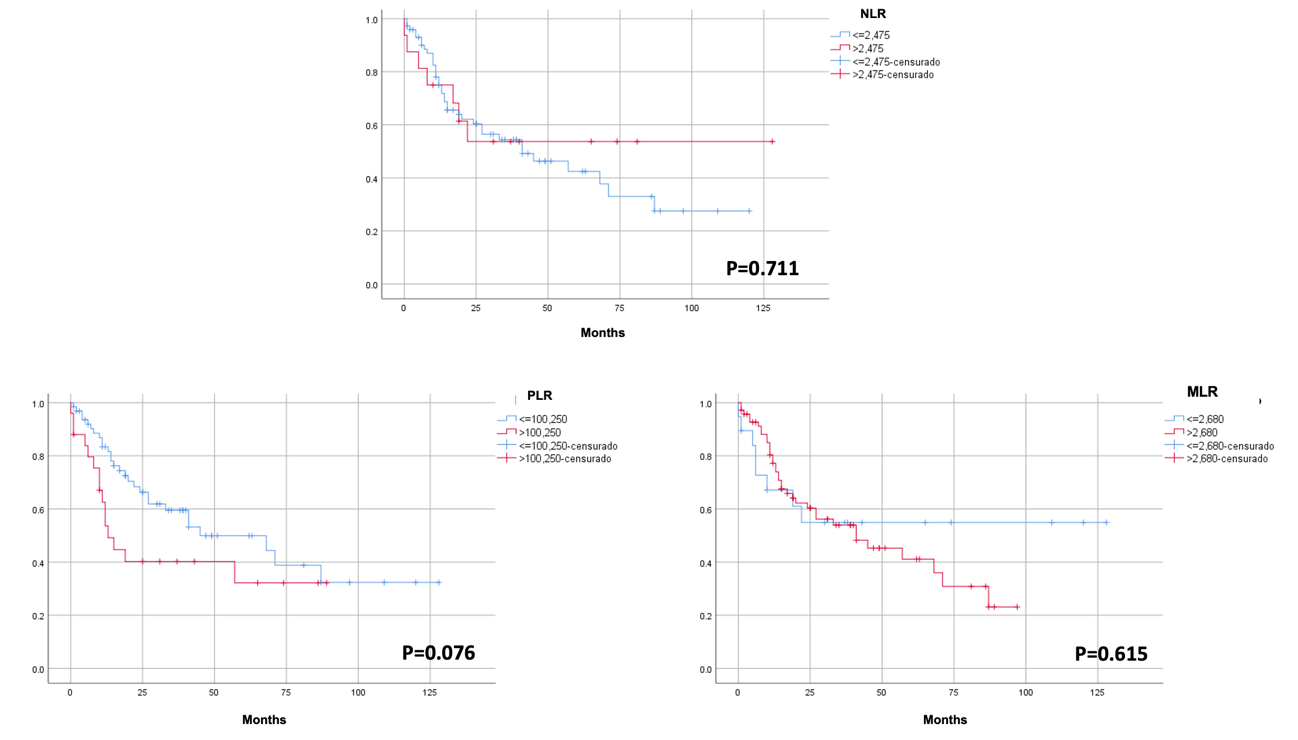


**Figure S3.** Disease-free survival of patients with hepatocellular carcinoma < 5 cm (Group 1) with low (blue) and high (red) neutrophil-to-lymphocyte ratio (NLR), platelet-to-lymphocyte ratio (PLR), and monocyte-to-lymphocyte ratio (MLR)


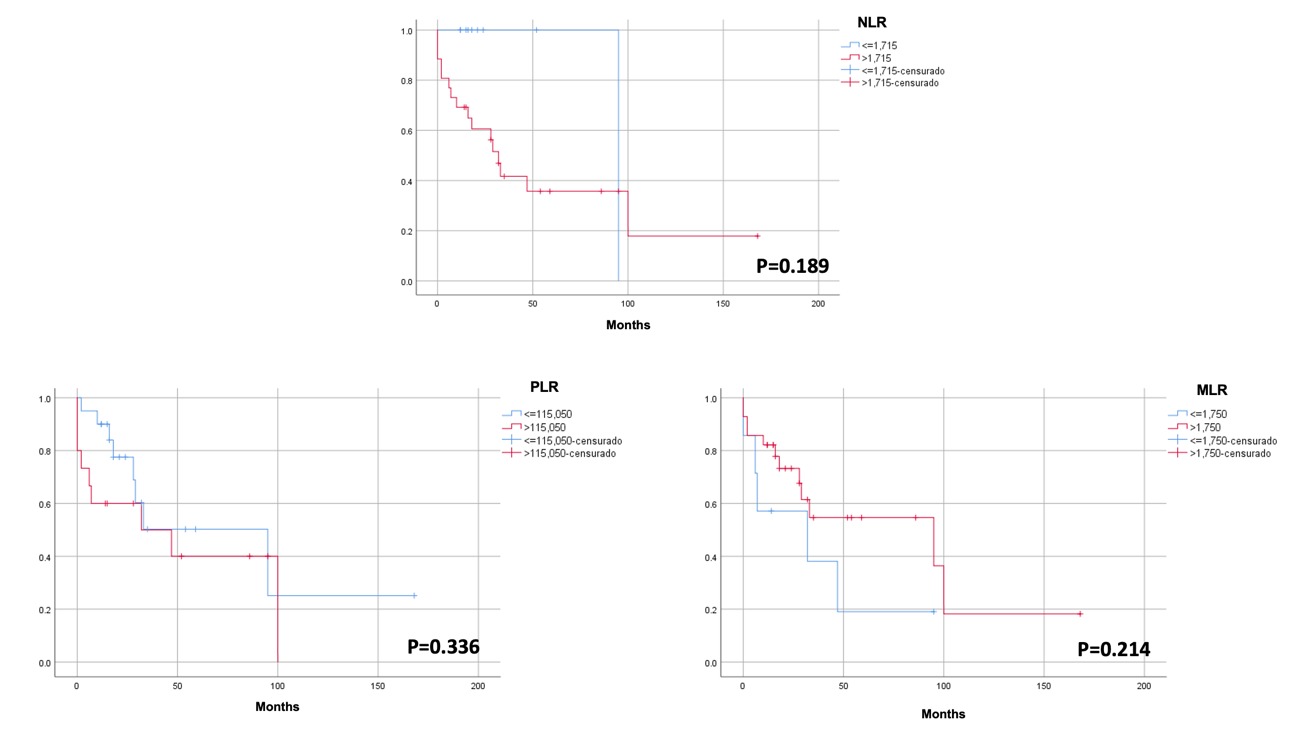


**Figure S4.** Overall survival of patients with hepatocellular carcinoma between 5 and 10 cm (Group 2) with low (blue) and high (red) neutrophil-to-lymphocyte ratio (NLR), platelet-to-lymphocyte ratio (PLR), and monocyte-to-lymphocyte ratio (MLR)


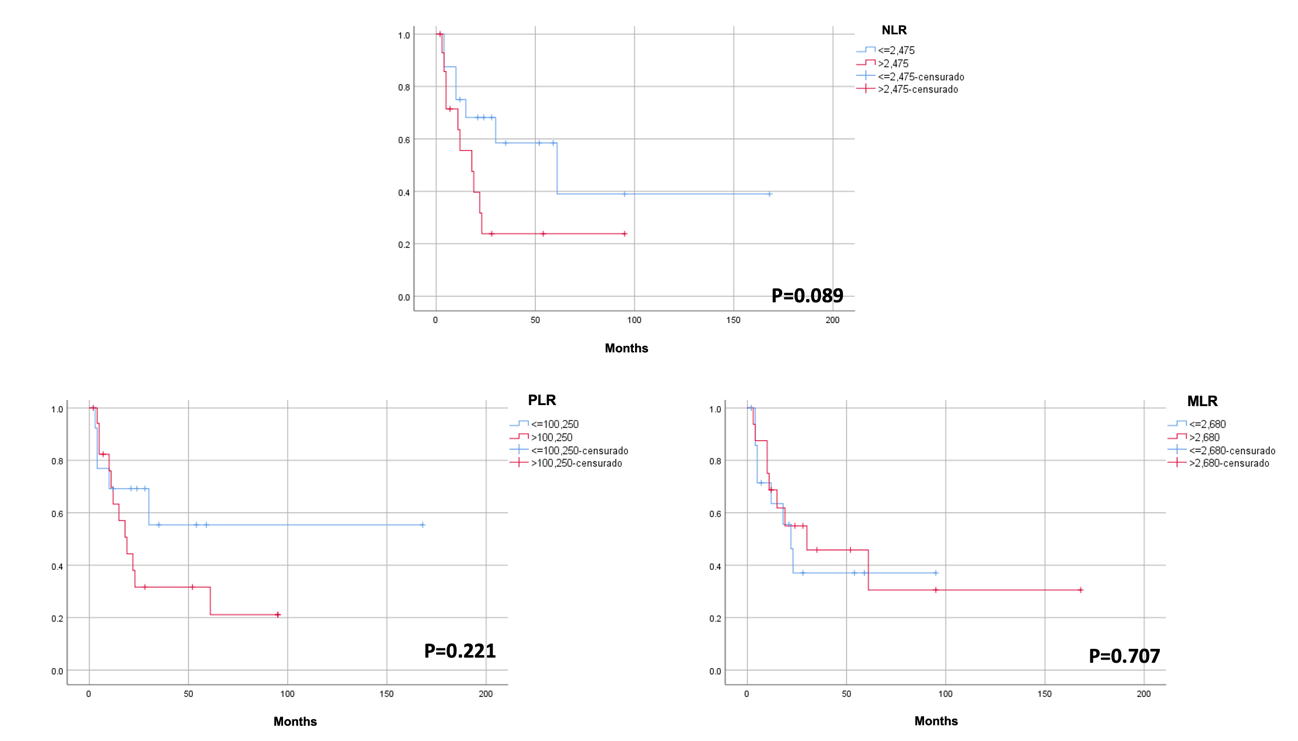


**Figure S5.** Disease-free survival of patients with hepatocellular carcinoma between 5 and 10 cm (Group 2) with low (blue) and high (red) neutrophil-to-lymphocyte ratio (NLR), platelet-to-lymphocyte ratio (PLR), and monocyte-to-lymphocyte ratio (MLR)


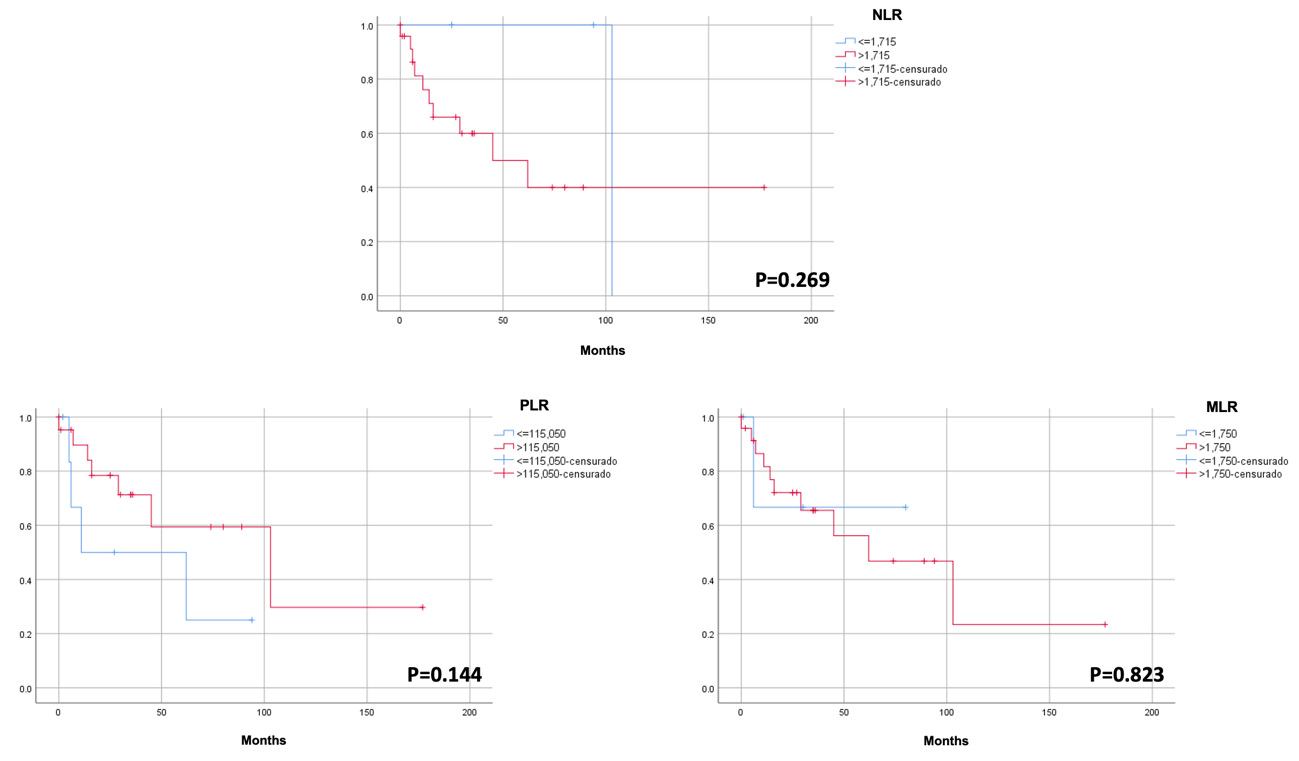


**Figure S6.** Overall survival of patients with hepatocellular carcinoma > 10 cm (Group 3) with low (blue) and high (red) neutrophil-to-lymphocyte ratio (NLR), platelet-to-lymphocyte ratio (PLR), and monocyte-to-lymphocyte ratio (MLR)


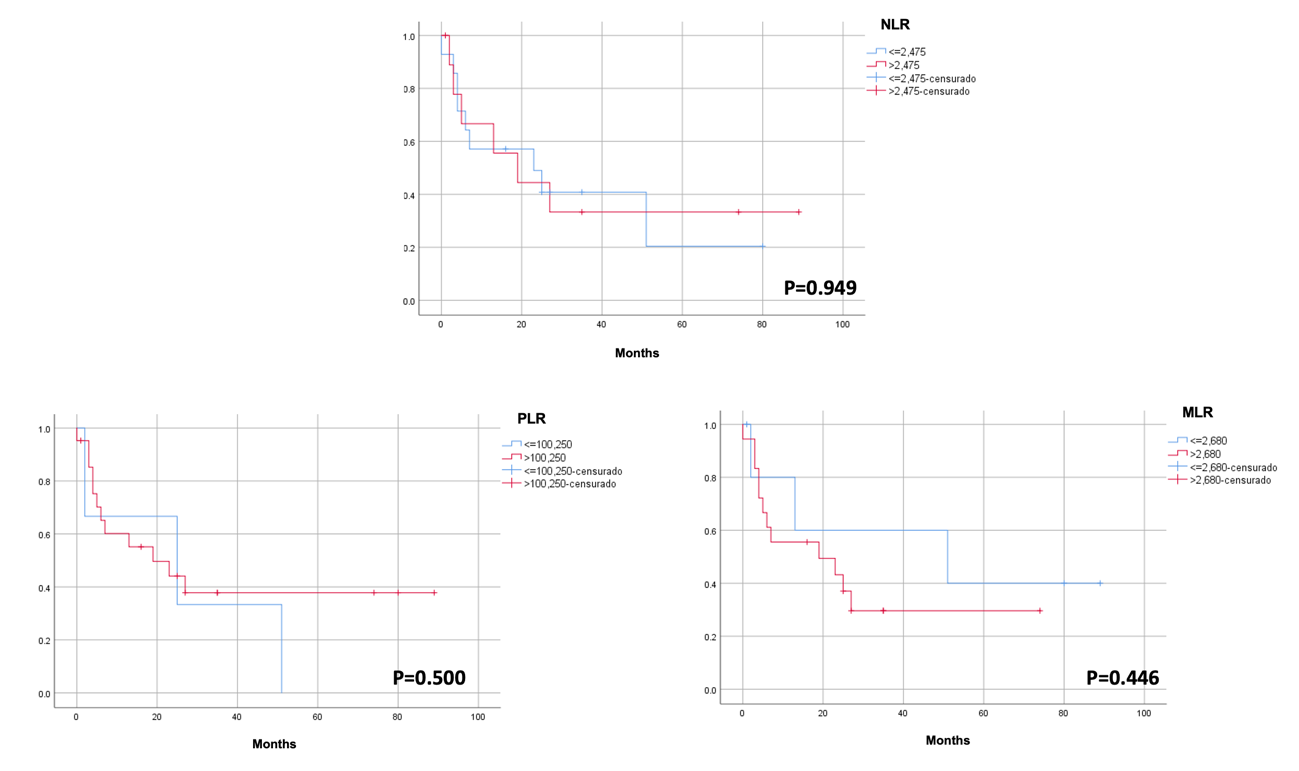


**Figure S7.** Disease-free survival of patients with hepatocellular carcinoma > 10 cm (Group 3) with low (blue) and high (red) neutrophil-to-lymphocyte ratio (NLR), platelet-to-lymphocyte ratio (PLR), and monocyte-to-lymphocyte ratio (MLR)
